# Structural and pragmatic language skills in school-age children relate to resting state functional connectivity

**DOI:** 10.1007/s11682-025-01040-7

**Published:** 2025-07-08

**Authors:** Julia C. Hoyda, Hannah J. Stewart, Jennifer Vannest, Karla N. Washington, David R. Moore

**Affiliations:** 1 University of Cincinnati, Cincinnati, USA; 2 Department of Psychology, University of Lancaster, Lancaster, UK; 3 University of Toronto, Toronto, Canada; 4 Division of Patient Services Research, Cincinnati Children’s Hospital Medical Center, Cincinnati, USA; 5 Manchester Centre for Audiology and Deafness, University of Manchester, Manchester, UK

**Keywords:** Structural language, Pragmatic language, Functional connectivity, RsfMRI

## Abstract

Language difficulties are common in school-age children but their etiology is often unknown. Although neural underpinnings of language have been well-studied in neurotypical individuals, functional connectivity differences between children with language difficulties and their typically-developing peers have not. There is little evidence regarding patterns of neural connectivity for children with language difficulties. Differences in neural networks related to degree of language difficulties and subtype of language skills (structural or pragmatic) are unclear. We examined expressive and receptive language networks, and an executive function network, in school-age children (8–12 years, *n* = 81) relative to their caregiver-reported language skills. We hypothesized that children with poorer structural and pragmatic language skills would have decreased connectivity in these networks. Participants were separated into groups by structural and pragmatic language scores: those with structural language difficulties (SLD), pragmatic language difficulties (PLD), and combined language difficulties (CLD, consisting of some participants in both SLD and PLD). The remainder of participants were in the typical language (TL) group. Results showed trends toward increased cross-hemispheric connectivity in age-matched controls relative to those with poorer language skills. Specifically, connectivity between bilateral inferior frontal gyri and areas including bilateral supplementary motor areas, cerebellar regions, and bilateral frontal gyri was associated with higher structural and pragmatic language scores. Connectivity among additional regions including bilateral superior temporal gyri and Heschl’s gyrus showed both positive and negative correlation with both language scores. This suggests that reduced connectivity between regions involved in language processing may contribute to language difficulties in school-age children.

**Supplementary Information:**

The online version contains supplementary material available at 10.1007/s11682-025-01040-7.

## Introduction

Variability in language skills, including receptive and expressive language difficulties, is well-documented in school age children. When children exhibit difficulties with speech and language, those difficulties often associate with life-long difficulties with social communication, reading, and other skills that have academic, occupational, and social consequences (HHS, 2022). Behaviorally, a longitudinal population study by Reilly and colleagues revealed that the best predictors of language development (namely toddler communication and expressive vocabulary) include maternal education, SES, and a family history of speech and/or language difficulties (Reilly et al., 2009, 2018). The majority of those diagnosed with developmental language disorder (DLD) have difficulties with both expressive and receptive aspects of language (Calder et al., 2022).Language difficulties presumably have a neural basis in brain networks, but information about these specific neural mechanisms in school-age and younger children is limited. Understanding the neural basis of language difficulties during development could, in the long-term, have implications for targeted assessment and intervention.

Most of the literature on the neural basis of language, both in healthy individuals and individuals with language difficulties, is focused on adults. In this population, both lesion-based and neuroimaging data support the organization of cortical language circuits into multiple pathways in the left hemisphere; specifically, a ventral pathway involved in translating auditory input to meaning, and a dorsal pathway that translates auditory input to motor movements (Friederici, 2012; Hickok & Poeppel, 2007). The ventral pathway connects inferior frontal cortex with the temporal pole and with anterior and posterior temporal regions in the middle and superior temporal gyri/sulci. These connections are suggested to support processing of phonological, lexical, syntactic, and semantic information. The dorsal pathway connects the posterior superior temporal cortex to the premotor cortex via the inferior parietal cortex, and the superior temporal gyrus to the pars opercularis of the inferior frontal gyrus. The dorsal pathway has been suggested to support the mapping of phonological to motor representations and of speech sounds to words (Friederici, 2012; Hickok & Poeppel, 2007). Broadly, both ventral and dorsal pathways connect overlapping networks involving the inferior frontal gyrus, middle and superior temporal regions, including the insula (Zaccarella & Friederici, 2015), and temporo-parietal regions. These networks are simultaneously engaged during both expressive and receptive language functions (speech sounds, phonological processing, syntactic, and semantic processing).

From a developmental perspective, Hickok and Poeppel (2007) note that the dorsal pathway may be particularly engaged during language learning, and a number of other studies have focused on the maturation of neural networks supporting language skills during typical development. Differences between brain networks underlying language skills in individuals with and without speech and language difficulties are not as consistent or well-defined. Some recent studies have used structural and functional neuroimaging to observe differences in neural architecture and activation (Badcock et al., 2012; de Guibert et al., 2011; Dehaene-Lambertz et al., 2002; Dibbets et al., 2006), primarily showing differences in regions that are part of the dorsal or ventral pathways, though not distinguishing between the pathways. For example, Badcock et al. (2012) examined neural structure and function in children with Specific Language Impairment (SLI)^1^, their typical siblings, and another, typical but unrelated control group, all ranging in age from 6 to 25 years old. They found that the participants with SLI had decreased gray matter compared to the control groups in areas including bilateral superior temporal cortices and the right caudate nucleus, but increased gray matter in the left IFG. This contrasts with findings from Preston et al. (2014) who found children with speech sound errors had higher gray matter volume in the STG bilaterally. Plante et al. (1989) examined a set of 5-year-old dizygotic twins, one of whom had SLI. They found that the twin with SLI had bilateral volume symmetry in the perisylvian areas, whereas the twin without SLI had a slightly larger right perisylvian area. A review by Ullman et al. (2024) revealed that among participants with developmental language disorder (DLD), there are structural abnormalities of the anterior portion of the basal ganglia in both hemispheres. The anterior portion of the basal ganglia is responsible for procedural learning and memory which supports grammar and language processing, and differences in its structure along with other areas in the procedural learning circuit may lead to DLD (Janacsek et al., 2020; Tagarelli et al., 2019; Ullman, 2004; Ullman et al., 2020; Ullman & Pierpont, 2005).

Differences between children with SLI and typical controls in regional activation using functional MRI have also been observed. Dibbets et al. (2006) used a non-reading, rule-reversal task-switching paradigm, and found that children with SLI had increased activation in brain areas normally thought to be responsible for executive function, including cingulate and frontal regions (Dibbets et al., 2006). The lateralization of language processing continues through childhood (Enge et al., 2020; Kennedy-Higgins et al., 2020; Plante et al., 2015), though when this lateralization peaks is still debated (Holland et al., 2007; Karunanayaka et al., 2007; Szaflarski et al., 2006; Weiss-Croft & Baldeweg, 2015). de Guibert and colleagues (2011) examined lateralization of activation during naming and phonological tasks where children with SLI showed significantly less left lateralization in the IFG pars opercularis, pars triangularis, STG, and supramarginal gyrus (SMG). Specifically, children with SLI had decreased activation in the left STG/SMG during the naming task, and increased activation in the anterior insula and caudate nucleus while performing a phonological task. Partially consistent with these results, Badcock et al. (2012) found that, during a naming task, the participants with SLI exhibited decreased activation in the bilateral superior temporal cortices, the left IFG, and right putamen. Relatedly, during encoding and recognition tasks, Ellis Weismer et al. (2005) found that a group of adolescents with SLI had reduced activation compared to typical peers in the IFG, precentral sulcus, and a posterior region in the left parietal lobe, and overall an inconsistent activation pattern of regions in the left hemisphere during an encoding task. In contrast, Krishnan et al. (2021) compared a large sample of children with developmental language disorder (DLD) with typically developing peers on a verb generation task and found no group differences in activation in the left IFG or in lateralization within language regions. Overall, the findings from these studies show that individuals with language difficulties frequently had reduced activation within the IFG, superior temporal gyri, and insula, compared to typical controls, but varying hypo- or hyperactivation in subcortical areas such as the putamen and caudate nucleus (Badcock et al., 2012; de Guibert et al., 2011; Dibbets et al., 2006; Ellis Weismer et al., 2005). Some studies also reported different patterns of hemispheric asymmetry (Badcock et al., 2012; de Guibert et al., 2011; Plante et al., 1989).

Most studies that have examined speech and language skills in children, including those analyzing their neural substrates, have focused solely on “structural” language skills, specifically phonology, morphology, and syntax. These skills have also been the focus of studies on the dorsal and ventral pathways (Friederici, 2012; Hickok & Poeppel, 2007). However, pragmatic language skills, such as initiation of conversation or use of social language, are crucial to communication development, and are a known area of impairment (Fujiki et al., 2004; Hart et al., 2004; Redmond, 2011; Spackman et al., 2006). Pragmatic language skills involve understanding a social context, incorporating real-world knowledge, and processing emotional content and context of language, among others (Cummings, 2021; Johnson et al., 1984). These skills are most often examined only in relation to autism spectrum disorder (ASD) and/or behavioral difficulties. Although children will often exhibit deficits across both sets of skills, many children will have intact structural language skills while showing impaired pragmatic language skills, as often found in children with ASD and emotional/behavioral difficulties (Law et al., 2013; Mackie & Law, 2010; Reindal et al., 2021; Volden & Phillips, 2010). Deficits in pragmatic language skills can preclude children from developing proper structural language skills. Because the social brain learns speech through mimicry and interlocutors, those with pragmatic difficulties have an extra hurdle to cross (Kuhl, 2007). Pragmatic factors of maternal mental health, namely degree and type of social interaction with the child, affect the onset and offset of critical periods for speech and language development (Werker & Hensch, 2015). Other pragmatic components such as sustained joint attention, shared environment, and regular communication routines (Bruner & Watson, 1983) provide scaffolding for language development (Hirsh-Pasek et al., 2015; Romeo et al., 2018; Tamis-LeMonda et al., 2014; Vygotskiĭ & Cole, 1978; Zimmerman et al., 2009). Conversational turn-taking is also positively correlated with vocabulary growth (Donnelly & Kidd, 2021). Conboy et al. (2015) show that the greater an infant’s ability is to exhibit social responses, the greater the degree that child will learn phonemes and words.

Deficits in pragmatic language skills are not unique to children with ASD. Children from families of low socio-economic status may struggle with both structural and pragmatic language (Law et al., 2013). Particularly for these children, pragmatic language skills have been shown to be an arbiter of behavioral difficulties and broader language difficulties (Law et al., 2013). Another study found that 94% of children who were referred to specialists for behavioral difficulties had difficulties in structural as well as pragmatic language skills (Mackie & Law, 2010).

There is limited data on the neural basis of pragmatic language, and most studies are in adults. Kuperberg and McGuire (2000) used fMRI in typical adults and found that, when contrasting normal sentences and sentences with pragmatic, semantic, and syntactic violations, the pragmatic violation condition elicited unique activation of the left STG compared to the semantic and syntactic violation conditions. Basnakova et al. (2014) used a task requiring participants to infer meaning from a spoken sentence; sometimes the meaning was direct, other times it was implied or not verbally apparent (indirect). When comparing direct and indirect conditions, they discovered higher activation in the bilateral IFG, right middle temporal gyrus, and right temporo-parietal junction during the indirect condition.

Overall, these neuroimaging studies of both structural and pragmatic language included a range of populations (Badcock et al., 2012; de Guibert et al., 2011; Dibbets et al., 2006; Plante et al., 1989; Preston et al., 2014), and relatively few have been conducted in school-age children, when language disorders are prevalent and most often diagnosed (McGregor et al., 2020). Models of the neural basis of language supporting these studies focus strictly on structural aspects of language, like syntax and semantics, and do not consider pragmatic language skills (Dibbets et al., 2006; Ellis Weismer et al., 2005). Although the studies have examined the structure and activation of language regions via a variety of language tasks, relatively little is known about patterns of connectivity for children who have language difficulties and how neural networks are related to types and levels of language skills in these children. This information can provide insights about temporal synchrony among language processing regions.

Most of the reviewed studies report on task-based fMRI activation. In contrast, resting state connectivity examines regional, network functional associations that are highly temporally correlated (Biswal et al., 1995). The spontaneous neural activity that is picked up by resting state fMRI is correlated with activation of known regions and functions (i.e. auditory and visual processing), and is not due to motion or other artifacts (Cordes et al., 2000; Lowe & Sorenson, 1998; Stein et al., 2000; Xiong et al., 1999). Resting state imaging generalizes well to the study of language because activity across regions in dorsal and ventral pathways has been shown to be correlated during the resting state, perhaps indicating a cooperative interaction for language processing (Power et al., 2011, 2014). Extensive data from multiple systems showing which regions of the brain co-activate at rest has provided insight into how neural networks are organized and work in concert, and how they may be disrupted in individuals who have difficulty with specific skills, such as structural or pragmatic language (Cordes et al., 2000; Cross et al., 2021; Hampson et al., 2002; Huang et al., 2024; Xiong et al., 1999).

Pragmatic language skills require the integration of expressive and receptive language with social cognition and higher-level cognitive skills such as initiation and control and so have been proposed to depend highly on executive function (Andres-Roqueta et al., 2021), especially in those with ASD (Razavi et al., 2019). However, pragmatic language and executive function also have a relationship in those with ADHD (Crisci et al., 2022). The Flanker Test, which was developed from the Attention Network Test (Rueda et al., 2004), and the Dimensional Change Card Sort (DCCS) Test are part of the NIH toolbox and reliably measure executive functioning skills (Weintraub et al., 2013; Zelazo, 2006). Even though these tests are not language-specific, pragmatic language is dependent on executive function so the tests are useful measures informing relationships between the two sets of skills.

The current study was conducted across a group of school-age children with variable language skills, measured by the Children’s Communication Checklist-2, or CCC-2 (Bishop, 1998). The CCC-2 is a parent-reported measure of the child’s speech and language abilities across ten categories. In a study by Volden and Phillips (2010) in children with ASD, the CCC-2 was superior to other tests in detecting pragmatic language difficulties. The CCC-2 also has a 70% sensitivity and an 85% specificity for SLI (Bishop, 2003) and differentiates between structural and pragmatic language skills, separating the two into different sets of subscales.

In addition to language networks, we examined functional connectivity in an executive network due to the dependency of pragmatic language skills on executive function (Andres-Roqueta et al., 2021; Crisci et al., 2022; Razavi et al., 2019). Our aims were to (1) document the profiles of structural and pragmatic language skills in a group of school-age children using the CCC-2 and (2) relate these to patterns of functional connectivity in the selected brain networks. We hypothesized that children with poorer structural and pragmatic language skills would demonstrate decreased connectivity in these language and executive function networks. Specifically, we expected a negative correlation between pragmatic language skills and connectivity in the executive and language networks and a negative correlation between structural language skills and connectivity in speech networks.

## Methods

*Participants*: Study data were from 81 children (54 male and 27 female; mean age 9.53 years, SD 1.9) recruited as part of a larger, longitudinal investigation of listening difficulties (166 enrolled children, *R01DC014078*,* Moore*,* PI*) at Cincinnati Children’s Hospital Medical Center. Recruitment was via social and digital media messages as well as print advertising at the Hospital. Participants were recruited based on a suspicion of listening difficulties (or auditory processing disorder). Medical records were reviewed for diagnoses of auditory processing disorder or auditory processing “weaknesses” (details of these diagnoses and terms are found in Moore (2018). Parent/caregiver questionnaires included the Children’s Communication Checklist-2 (CCC-2) and the Evaluation of Children’s Listening and Processing Skills, or ECLiPS (Barry & Moore, 2021; Denys et al., 2024; Petley et al., 2021). Participants were recruited through advertisements for children with everyday listening difficulties, and then screened via a caregiver ECLiPS questionnaire regarding everyday listening skills. Other relevant medical and demographic data (i.e. maternal education level) were used for participant selection and analysis. 28 caregivers reported their child having seen a speech-language pathologist. Of these, 11 reported that they received a diagnosis, though the specific diagnosis was not collected in this report. 8 reported that they did not receive a diagnosis, and the remainder did not answer. One participant in the pragmatic language difficulties group (see below for group details) had a diagnosis of ASD. One participant in the combined language difficulties group had a diagnosis of PDD-NOS and one participant in the typical language group had a diagnosis of PDD. Full details of inclusion and exclusion criteria are reported elsewhere (Petley et al., 2021). All children participated in an audiology screening and had normal pure tone thresholds of *≤* 20 dB HL at all octave-interval frequencies from 0.25 to 8 kHz in both ears. The children’s auditory abilities (Hunter et al., 2021; Petley et al., 2021) brainstem (Hunter et al., 2023; Petley et al., 2024) and auditory cortical function (Moore et al., 2020; Stewart et al., 2022) are reported elsewhere. Maternal education level was also collected via caregiver-report.

*Caregiver report language measure*: Caregivers of all participating children completed the CCC-2, an instrument used to assess the child’s skills in the following standardized scales: (A) Speech, (B) Syntax, (C) Semantics, (D) Coherence, (E) Initiation, (F) Scripted Language, (G) Context, (H) Nonverbal, (I) Social Relations, and (J) Interests. Each scale is standardized to a scaled score of 10 and a standard deviation of 3. From these scales we calculated a structural language score (averaged score from scales A, B, C, and D) and a pragmatic language score (averaged from scales E, H, I, and J). This allowed us to separate the structural and pragmatic language profiles of our participants, as in previous studies (Bishop, 1998; Timler, 2014), and relate those to resting-state functional connectivity data. We note that, in clinical use, structural and pragmatic language scores on the CCC-2 may be compared using a difference score called the Social Interaction Difference Index (SIDI). However, our focus was on structural and pragmatic abilities separately, rather than the difference between them. Participants were classified into four groups based on these structural and pragmatic language scores. Children with both structural and pragmatic language scores ≥ 7 comprised the typical language skills group (TL). Those with structural language score < 7 were included in the structural language difficulties group (SLD) and those with pragmatic scores < 7 were included in the pragmatic language difficulties group (PLD). Finally, children with scores < 7 in both the structural and pragmatic language scores made up the combined language difficulties group (CLD). This means that a participant might fall into and be represented in two groups (i.e. both the SLD and CLD groups). The groups were organized in this way because we hypothesized that each type of language difficulty (structural and pragmatic) has a specific neural basis. Therefore, we expected that a child that has both types of language difficulty would have evidence of both neural bases when compared to children with typical language skills, so these children were included in both the SLD and PLD groups). Examining the CLD group separately allowed us to explore whether there were differences in connectivity unique to having both types of difficulties, perhaps representing a more severe phenotype.

*Executive function assessment*. Also, as part of the larger study of listening difficulties, children completed the NIH Toolbox (Weintraub et al., 2013). In order to characterize the executive function skills of the children in our sample, we examined scores on two subtests from the NIH toolbox (the Flanker test and the Dimensional Change Card Sort test) for potential differences in executive function between the TL and LD groups. We also examined participants’ NIH cognition composite scores, which includes both the Flanker and Card Sort scores among other subtests.

*Functional Magnetic Resonance Imaging.* All children underwent a 5-minute resting state fMRI (TR/TE = 2000/30ms, voxel size = 2.5 × 2.5 × 3.5 mm, 39 axial slices, 150 volumes) in a 3T Philips Ingenia scanner with a 64-channel head coil. Children were told to hold still and focus on a fixation cross in the middle of a screen. All participants were awake and not sedated. The children also completed a T1-weighted anatomical scan (TR/TE = 8.1/3.7 ms, FOV = 25.6 × 25.6 × 16.0 cm, matrix = 256 × 256, and slice thickness = 1 mm). Each child’s scans were performed in a single scanning session at Cincinnati Children’s Hospital. Further details of the complete scanning protocol are included in Stewart et al. (2022).

### Data analysis

After categorizing the participants into groups as described above, we examined descriptive statistics for the CCC-2 subscale scores in each group, and the relationship between CCC-2 subscales. As a measure of socioeconomic status and its influence on language skills, we used a Pearson correlation to examine the relationship between maternal education and structural and pragmatic scores.

Statistical analyses of imaging data were completed using a general linear model (Nieto-Castanon, 2020) implemented in the Conn toolbox (Whitefield-Gabrieli & Nieto-Castanon, 2012). We examined differences in resting state functional connectivity associated with structural and pragmatic language skills, using three group comparisons. First, we compared the TL group to the SLD group. We then compared the TL group to the PLD group. Finally, we compared the TL group to the CLD group. Additionally, we examined relationships between functional connectivity and structural and pragmatic language scores as continuous variables. In each statistical model, age was included to account for developmental effects on functional connectivity. Maternal education was also included as a covariate in each model due to its influence on speech and language development (Aram & Levin, 2001; Dollaghan et al., 1999; Hoff, 2003).

#### Connectivity analyses

**Fig. 1:**
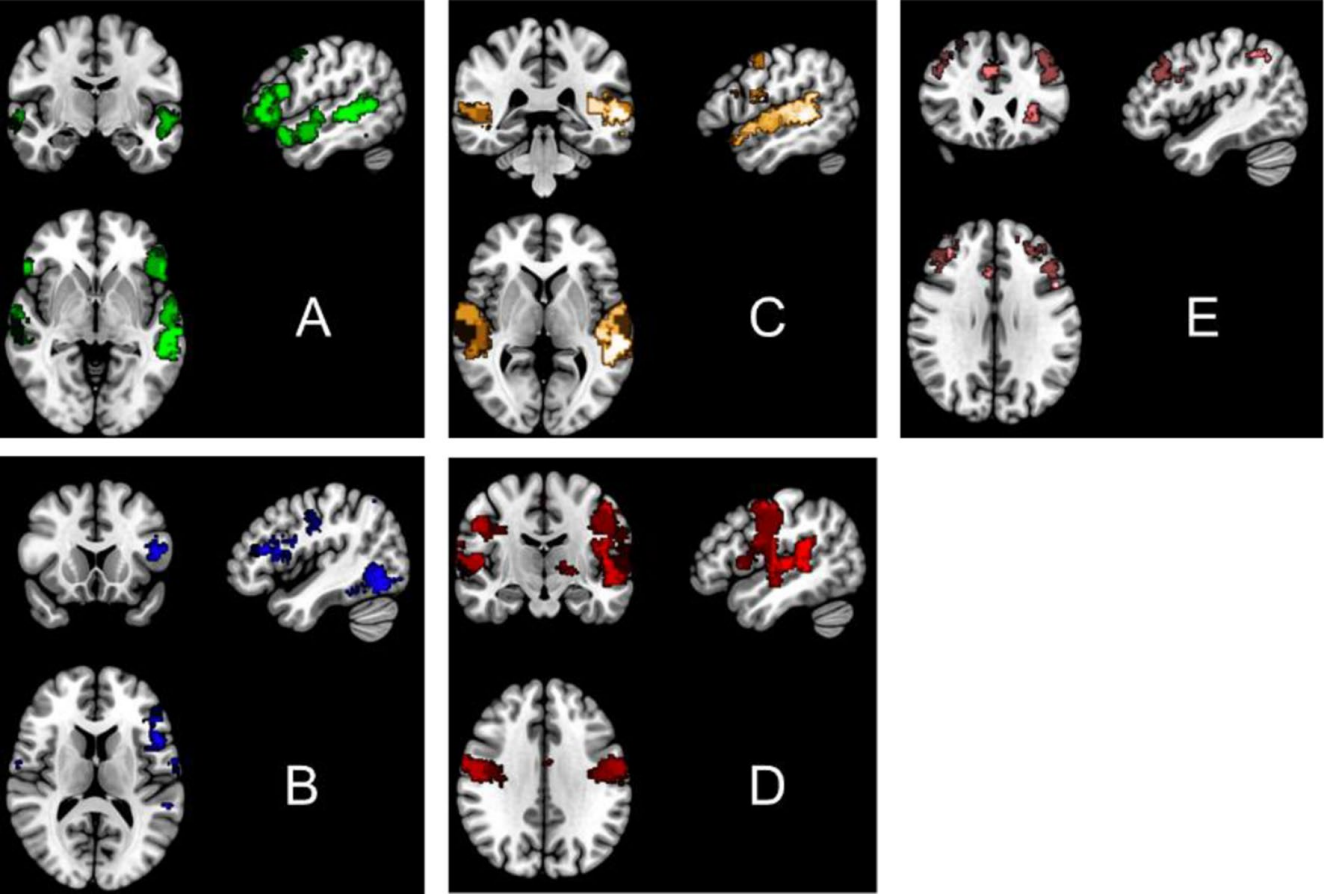
Slice view (R = L) of the networks used as inputs to connectivity analysis as determined by key term searches in *Neurosynth*. (**A**) Language Comprehension, (**B**) Naming, (**C**) Speech Perception, (**D**) Speech Production, (**E**) Executive. Note that because the networks based on *Neurosynth* spanned broad anatomic regions, these networks were parcellated into smaller ROIs based on subdivisions of the atlas by Bellec et al. (2017), in order to increase anatomical specificity and include ROIs of similar size in connectivity analyses

*A Priori Regions of Interest (ROIs) based on Neurosynth maps.* Regions of Interest (ROIs) were chosen from *Neurosynth.org*, an online meta-analysis of fMRI activation studies (Yarkoni et al., 2011). Neurosynth includes a broad database of thousands of adult and pediatric neuroimaging studies.The network maps thus produced encompass a broad scope of activation results based on search terms (which uses Activation Likeliness Estimation meta-analysis), each resulting in dozens to hundreds of studies represented by the map. In order to capture networks underlying multiple aspects of speech and language function, we chose four networks of ROIs using search terms ‘speech production’, ‘naming’ (for areas that contribute to expressive language), and ‘speech perception’ and ‘language comprehension’ (for areas that contribute to receptive language). These yielded networks that cover the regions of the dorsal and ventral language processing pathways, as can be seen in in Fig. 1 and anatomically described in Table 1. Notably, the structural scales of the CCC-2 incorporate both expressive and receptive aspects of speech and language skills, so we chose to examine a set of networks reflecting all of these language areas. In addition, given the association of pragmatic language skills and executive function (Andres-Roqueta et al., 2021; Crisci et al., 2022; Kapa & Plante, 2015; Razavi et al., 2019), we also examined an executive function network using the search term ‘executive’. While there are multiple networks engaged in EF, including the fronto-parietal network (containing bilateral IFG and MFG and the inferior and superior parietal lobules) and the cingulo-occipital network (containing regions such as dorsal anterior cingulate cortex and bilateral insula) (see Cacioppo et al. (1984); Collette et al. (2006); Congdon et al. (2011); Crittenden et al. (2016); Dosenbach et al. (2008); Dosenbach et al. (2007); Dosenbach et al. (2006); Menon and Uddin (2010); Nee et al. (2013); Niendam et al. (2012); Owen et al. (2005); Power et al. (2011); Thomas Yeo et al. (2011) for details of these regions). Neurosynth allowed a broad selection of regions that included the regions of these multiple networks (see Table 1 for the list of regions covered by this executive network from Neurosynth).

**Table 1:**
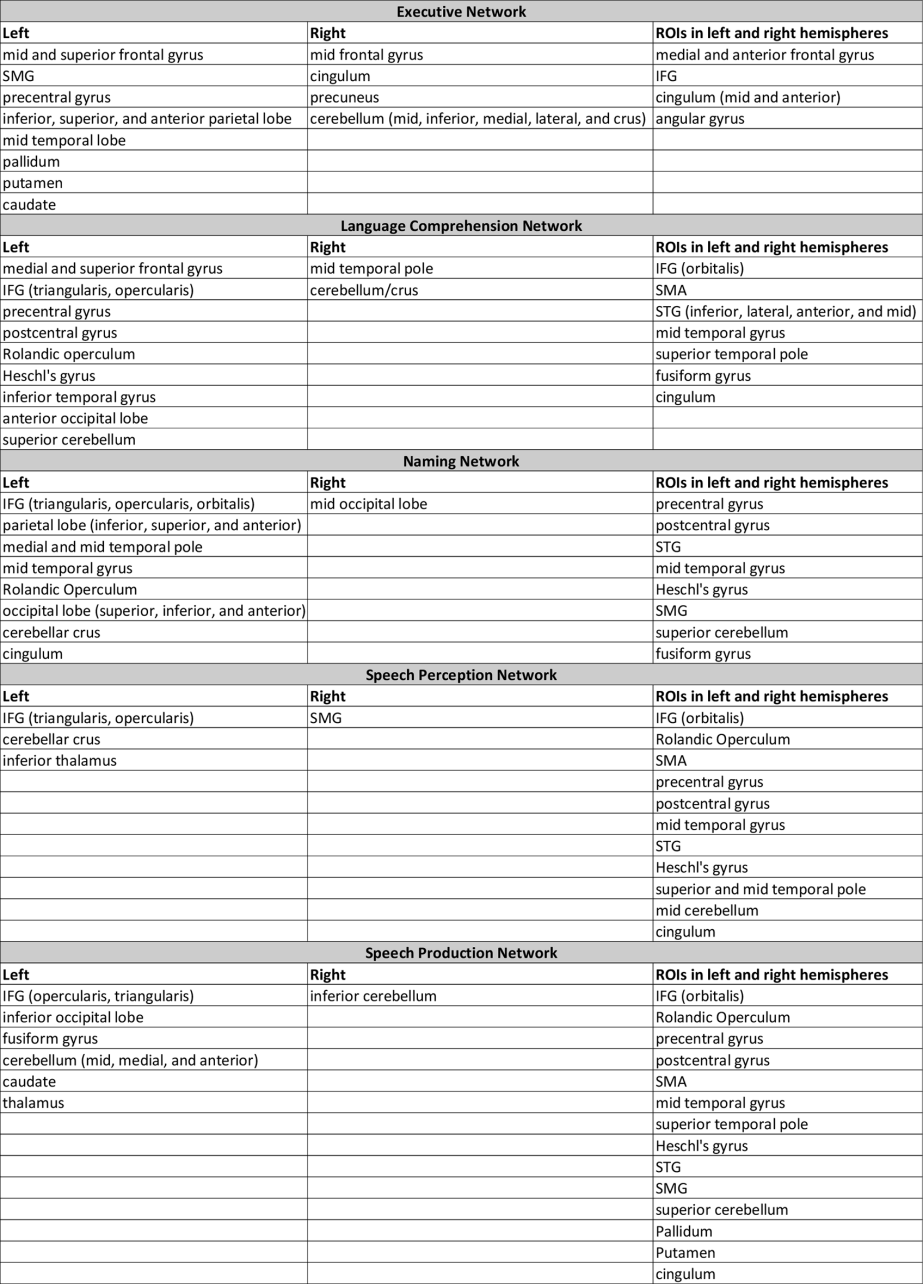
Anatomical descriptions of regions represented in each a priori network from neurosynth; separated by hemisphere

In order to construct networks composed of ROIs of approximately equal anatomic size, network maps from Neurosynth were parcellated using tools from FSL (Jenkinson et al., 2012)and the 200-region pediatric and young adult anatomical atlas developed by Bellec et al. (2017). Parcels 8 voxels or fewer in size were removed from analysis. This formed parcels that are appropriately sized for analysis. Varying ROI size can alter connectivity results; previous work supports keeping ROIs relatively similar in size (Etzel et al., 2009; Morabito et al., 2021; Poldrack, 2007; Wei et al., 2022; Zhu et al., 2012). The number of parcels in each network resulted as follows: ‘speech perception’ yielded 43 ROIs, ‘speech production’ 82, ‘language comprehension’ 54, ‘naming’ 56, and ‘executive’ 32.

*Computation of ROI-to-ROI connectivity.* rs-fMRI data was pre-processed using a standard pipeline (slice-timing correction, realignment, outlier identification, segmentation, normalization, and smoothing) in the Conn toolbox (Whitefield-Gabrieli & Nieto-Castanon, 2012). Data were realigned by each scan being aligned to a reference with the first image of the session.

The number of possible pairwise connections (non-directional) within each network is calculated by the equation $\:\left[\frac{n\left(n+1\right)}{2}\right]-n$, where n is the number of ROIs in the network. This means that with 43 ROIs, the speech perception network has a possible 903 pairwise connections. The speech production network has 3321 possible connections, the language comprehension network has 1431, the naming network has 1540, and the executive network has 496. Pairwise functional temporal connectivity was examined within each network of ROIs by taking the average time course of all voxels in each ROI. Using Fisher’s *z*-transformation, *r* values were converted to *z*-scores. These *z*-scores were used in group comparisons within each ROI network and in correlations with CCC-2 scores. Connectivity results were thresholded at a connection-level *p*<0.001, uncorrected. Multiple comparisons corrections (FDR) were applied at the cluster-level, with a threshold of *p* < 0.05. We also examined the results at connection level *p* < 0.005, uncorrected and cluster level *p* < 0.1, corrected (FDR) to look for trends in the data. These thresholds were applied to all results reported below. *Group comparisons.*

*Group comparisons.* Pairwise connectivity within each ROI network was examined for between-group comparisons. The connectivity group comparisons were made using Conn toolbox. Correction for multiple comparisons in each network was applied using false discovery rate (Benjamini & Hochberg, 1995).

*Relationships between connectivity and CCC-2 scores.* For every pairwise connection between the ROIs in each of the connectivity analyses, we examined the relationship between connectivity and CCC-2 structural and pragmatic scores across all participants using a Pearson correlation, to better understand how these connections related to children’s parent-reported language skills.

## Results

### *Profiles of language skills based on CCC-2*

**Fig. 2:**
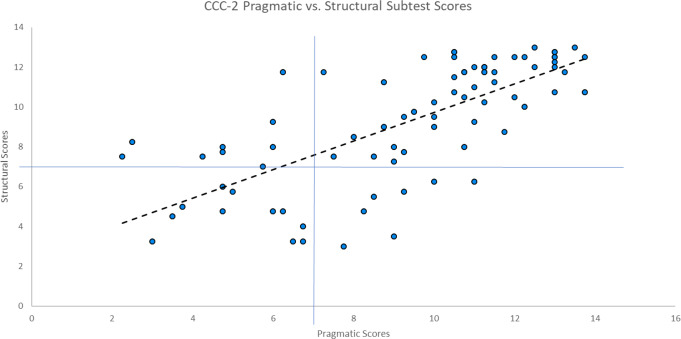
Scatterplot of CCC-2 Pragmatic language scores and Structural language scores. Blue horizontal and vertical lines indicate the “cut-off” score of 7 that was used to define groups (CLD, PLD, SLD, and TL). The diagonal line shows the linear correlation between the two sets of scores (df = 79, *r* = 0.715, *p* < 0.001)

Fifty-four children had both structural and pragmatic language scores on the CCC-2 that were in the typical range (age and sex scaled score *≥* 7, typical language [TL] group). Eighteen children evidenced structural language difficulties (scaled score < 7, SLD group) and 20 showed pragmatic difficulties (scaled score < 7, PLD group). Eleven children had scaled scores < 7 on both scales, showing combined language difficulties (CLD), and were included in both the structural and the pragmatic language difficulties groups. This left seven children uniquely in SLD and nine children uniquely in PLD. The eleven children that were in CLD were therefore, by definition, included in both the SLD and PLD groups. The structural and pragmatic scaled scores were positively correlated (Fig. 2; df = 79, *r* = 0.715, *p* < 0.001). The groups did not differ in maternal education or age (all *p* > 0.1). However, when examining maternal education as a continuous variable across all participants, maternal education was positively correlated with both structural (Rho = 0.356, *p* = 0.001) and pragmatic (Rho = 0.286, *p* = 0.01) language scores. DCCS scores differed between groups as follows: TL vs. PLD (unequal variances; Welch’s t = -4.3, df = 47.69, *p* < 0.001), TL vs. SLD (unequal variances; Welch’s t = -3.88, df = 46.29, *p* < 0.001), and TL vs. CLD (unequal variances; Welch’s t = -3.36, df = 20.1, *p* = 0.003). NIH cognition composite scores differed between groups as follows: TL vs. PLD (t = -4.789, df = 60, *p* < 0.001), TL vs. SLD (t = -4.984, df = 58, *p* < 0.001), and TL vs. CLD (t = -3.728, df = 53, *p* < 0.001). The cognition composite scores correlated both with the structural language scores (Pearson *r* = 0.638, *p* < 0.001) and the pragmatic language scores (Pearson *r* = 0.398, *p* < 0.001) as seen in Supplemental Table 1. See Table 2 for means and descriptive statistics of the groups.

**Table 2:** Mean (SD). Groups are determined by CCC-2 scores (see methods section)

	Typical Language	Pragmatic Language Difficulties	Structural Language Difficulties	^a^Combined Language Difficulties
n	54	20	18	11
Sex	34 M,20 F	16 M,4 F	13 M,5 F	9 M,2 F
Age	9.43 (1.93)	9.95 (1.96)	9.49 (1.82)	9.76 (2.02)
Maternal Education	3.96 (0.93)	3.25 (0.91)	3.61 (0.92)	3.27 (0.79)
CCC-2 structural scores	10.95 (1.67)	6.21 (2.28)	4.68 (1.1)	4.48 (0.96)
CCC-2 pragmatic scored	11.03 (1.62)	4.98 (1.4)	6.71 (2.32)	5.18 (1.36)
Card Sort*	100.95 (16.63)	86.2 (11.14)	88.07 (9.92)	87.11 (11.36)
Flanker*	97.43 (16.01)	91.73 (10.01)	91.41 (10.77)	93.4 (10.73)
Total cognition composite^b,^**	106.95 (17.31)	85.25 (11.25)	82.70 (12.75)	85.50 (11.44)

^a^Participants in the CLD group were also part of the PLD and SLD groups. Only seven children were unique to SLD and nine children unique to PLD. *TL*n* = 53, PLD*n* = 19, and SLD*n* = 17. **TL*n* = 45, PLD*n* = 17, SLD*n* = 15, CLD*n* = 10.^b^These scores encompassed flanker and card sort scores

When comparing the executive function skills of each group, as measured by the Flanker and Dimensional Change Card Sort tests, there were a few differences. The groups did not differ in their Flanker test scores (all *p* > 0.05). However, the TL group had higher Card Sort test scores than the other three groups (see Table 2).

### Group differences in connectivity

**Fig. 3:**
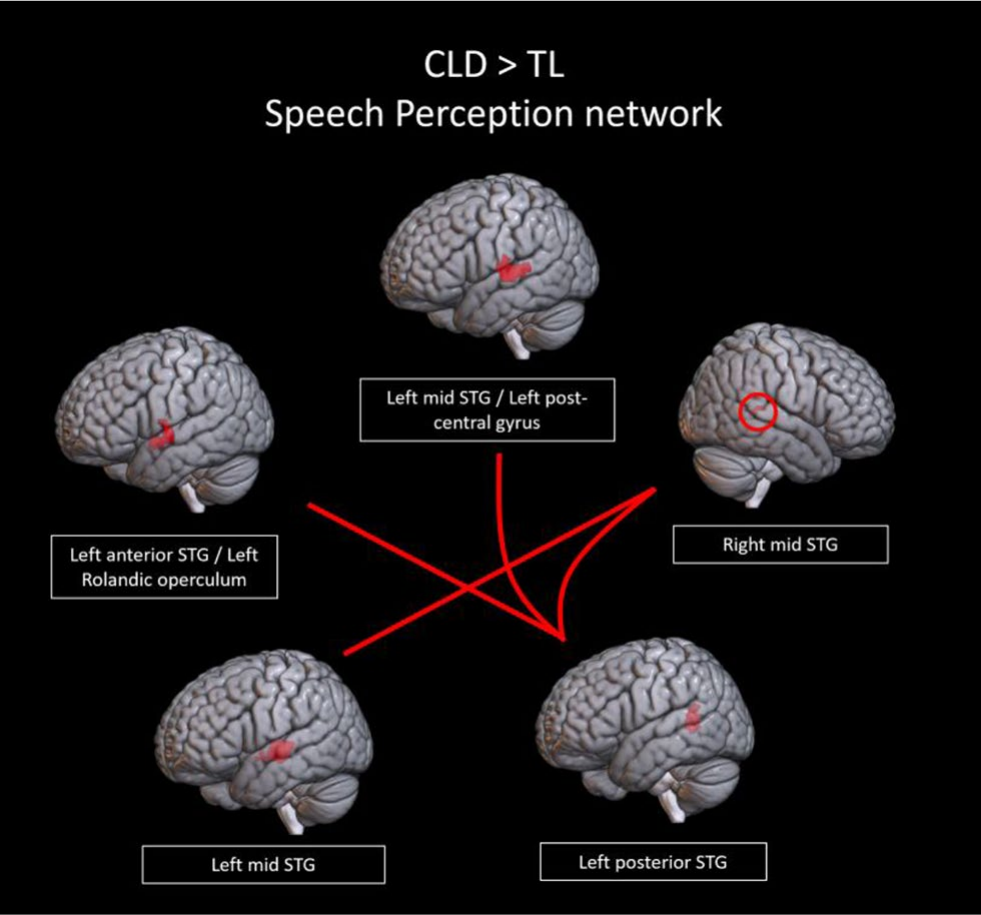
Connections between regions in the a priori speech perception network from neurosynth that showed a trend toward increased connectivity in the combined language difficulties group (CLD) compared to typical language peers (TL, connection *p* < 0.005, cluster *p* < 0.1)

*A priori ROI analysis based on Neurosynth.* There were no significant connectivity differences found between groups within the naming, language comprehension, speech production, or executive networks (all connection *p* > 0.005, all cluster *p* > 0.1). In the speech perception network, compared to the TL group, the CLD group exhibited a trend (connection *p* < 0.005, cluster *p* < 0.1) toward hyperconnectivity in connections between the left posterior STG and other regions including the right STG, left middle and anterior STG, and left Rolandic operculum. Another connection where the CLD group exhibited a trend toward hyperconnectivity was between the left and right mid STGs (Fig. 3).

### Relationships between connectivity and Language skills

*A priori ROI analysis based on neurosynth.* No significant correlations with CCC-2 structural or pragmatic scores as continuous variables were found with connectivity in the naming or speech perception networks.

**Fig. 4:**
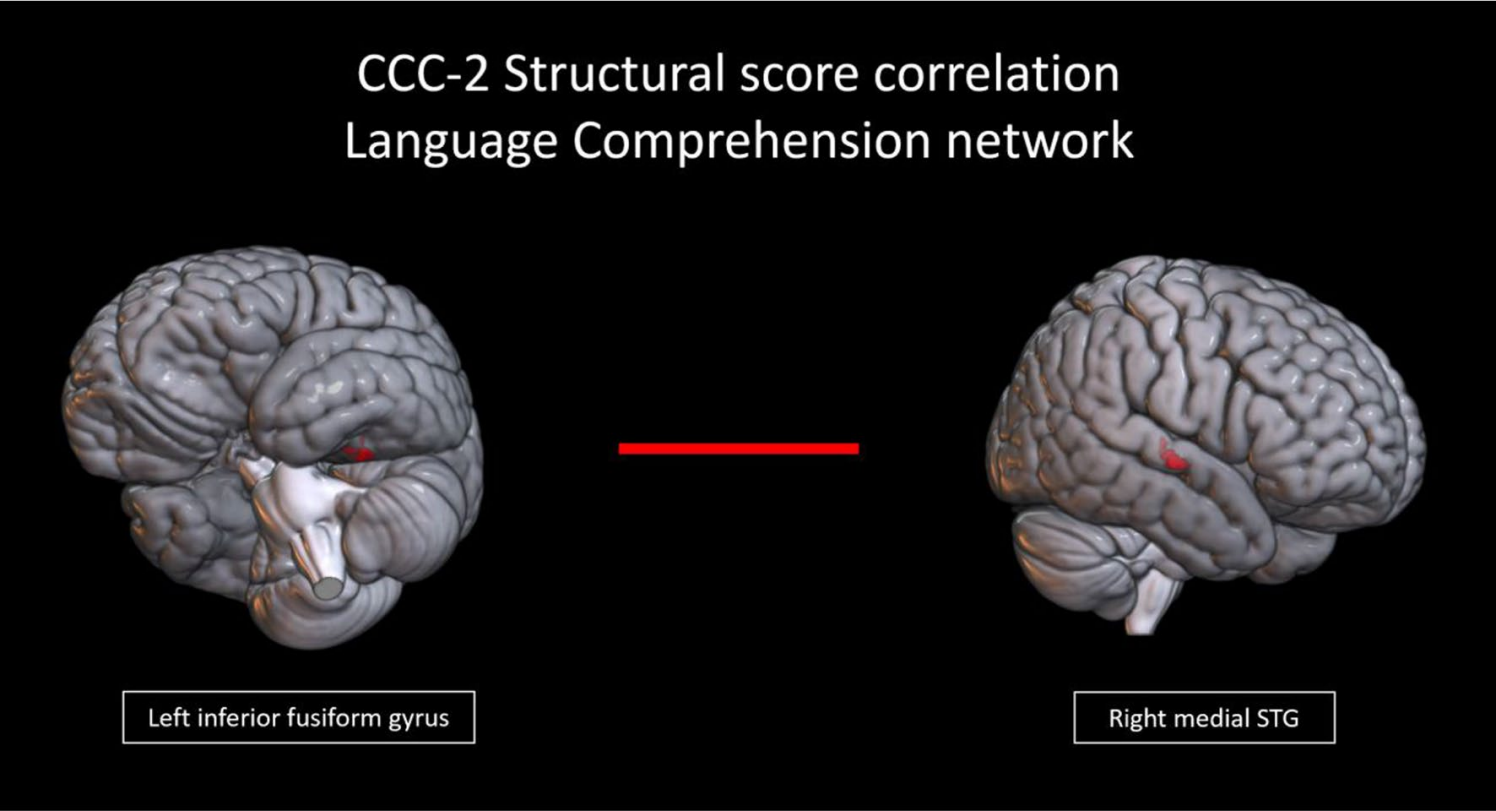
Connection between regions in the a priori language comprehension network from neurosynth that trended toward a positive correlation with CCC-2 structural scores (connection *p* < 0.001, cluster *p* < 0.1)

**Fig. 5:**
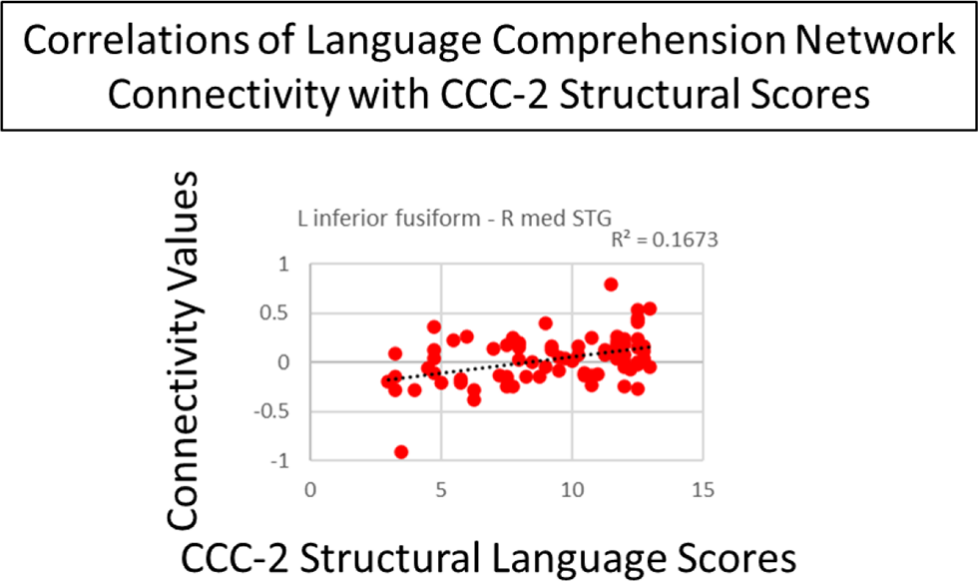
Scatterplot showing the correlation of the connectivity in the language comprehension network (connection between the left inferior fusiform and right medial STG) and CCC-2 structural language scores (all r^2^ displayed on graph, connection *p* < 0.001, cluster *p* < 0.1)

**Fig. 6:**
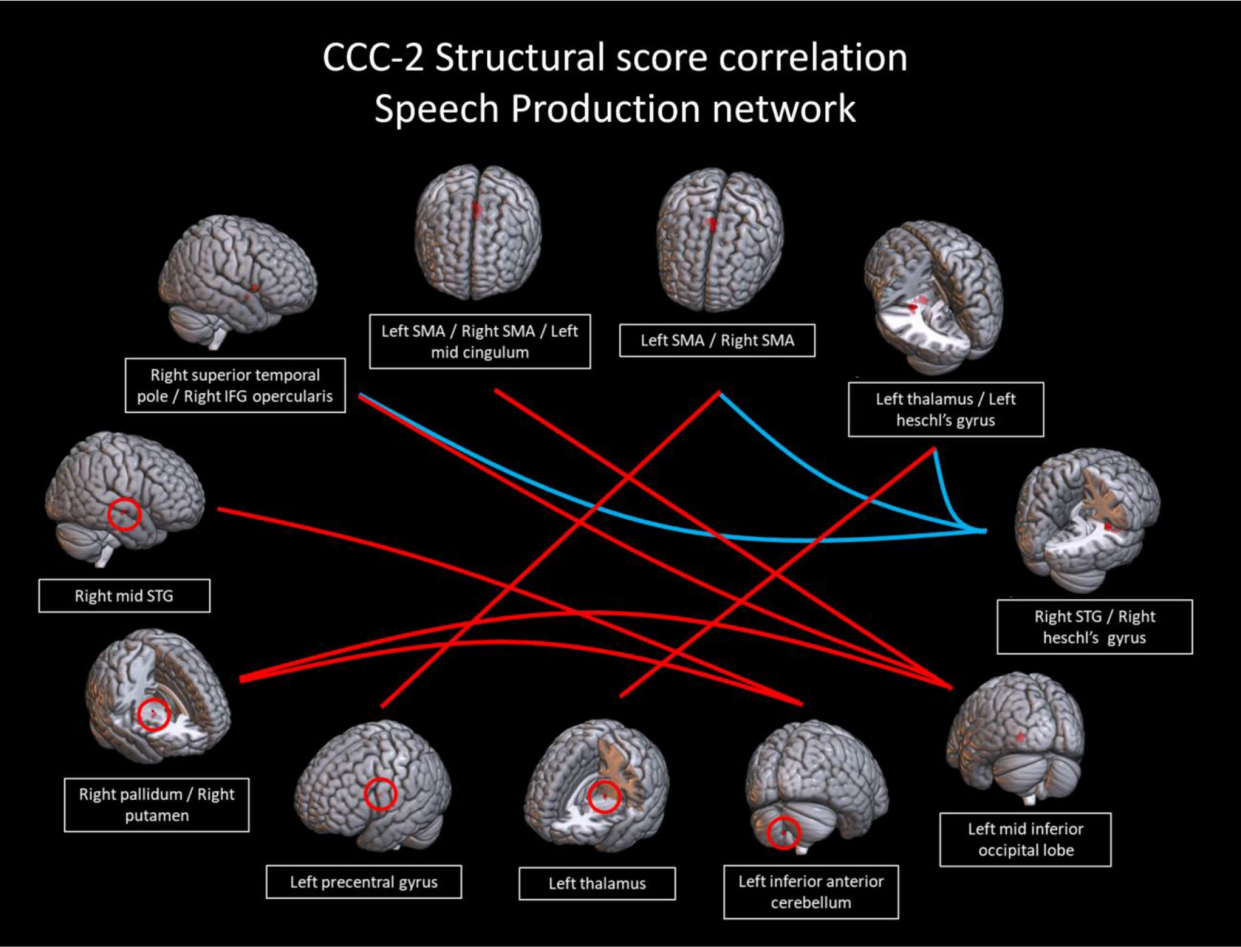
Connections between regions in the a priori speech production network from neurosynth; red connections were trends toward a positive correlation with CCC-2 structural scores; blue connections were trends toward negative correlations with the structural scores (connection *p* < 0.005, cluster *p* < 0.1)

**Fig. 7:**
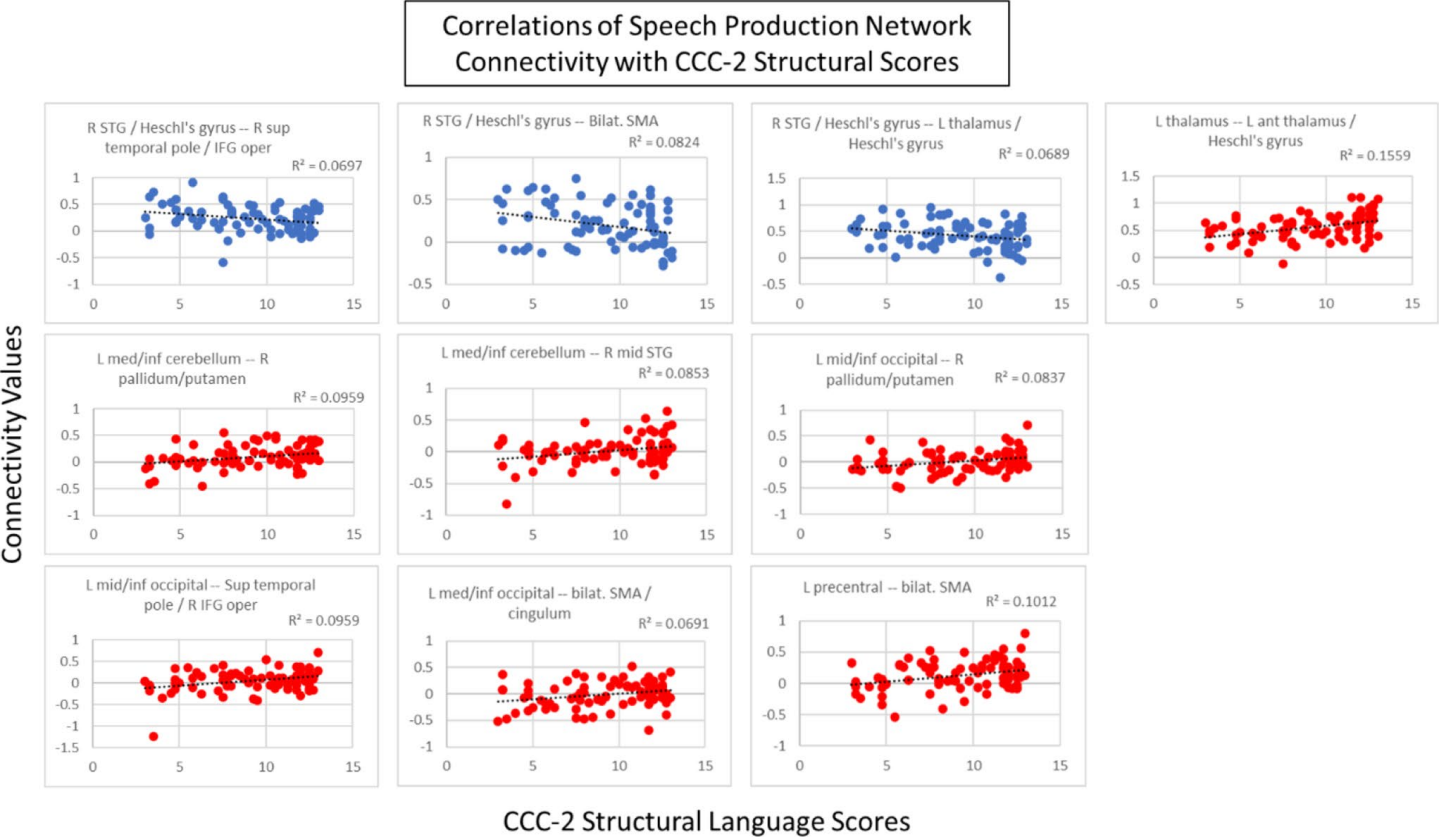
Scatterplots showing the correlation of the connectivity in the speech production network with CCC-2 structural language scores (all r^2^ values displayed on graph, connection *p* < 0.005, cluster *p* < 0.1). Blue shows negative correlations; red shows positive correlations

In the language comprehension and speech production networks, several connections showed a trend toward correlations with structural scores. In the language comprehension network, there was a trend (connection *p* < 0.001, cluster *p* < 0.1) toward a positive correlation with structural scores in a connection between the left inferior fusiform gyrus and right medial STG (Fig. 4). Figure 5 shows the correlations between connectivity and structural scores. In the speech production network, there was a trend (connection *p* < 0.005, cluster *p* < 0.1) toward a positive correlation with structural scores in cross hemispheric connections including those of the left mid inferior occipital lobe with (1) the bilateral medial supplementary motor areas (SMA), (2) the right superior temporal pole/right IFG pars opercularis, and (3) the right pallidum/putamen. Other connections that trended toward a positive correlation (connection *p* < 0.005, cluster *p* < 0.1) included the left inferior anterior cerebellum with (1) the right STG and (2) the right pallidum/putamen; as well as a connection of the left precentral gyrus with bilateral SMA; and a connection between the left thalamus and left Heschl’s gyrus. There was also a trend toward a negative correlation with structural scores in connections of right Heschl’s gyrus with (1) the left thalamus/left Heschl’s gyrus, (2) left/right SMA, and (3) the left cingulum (Fig. 6). Figure 7 shows the correlations between connectivity and structural scores.

**Fig. 8:**
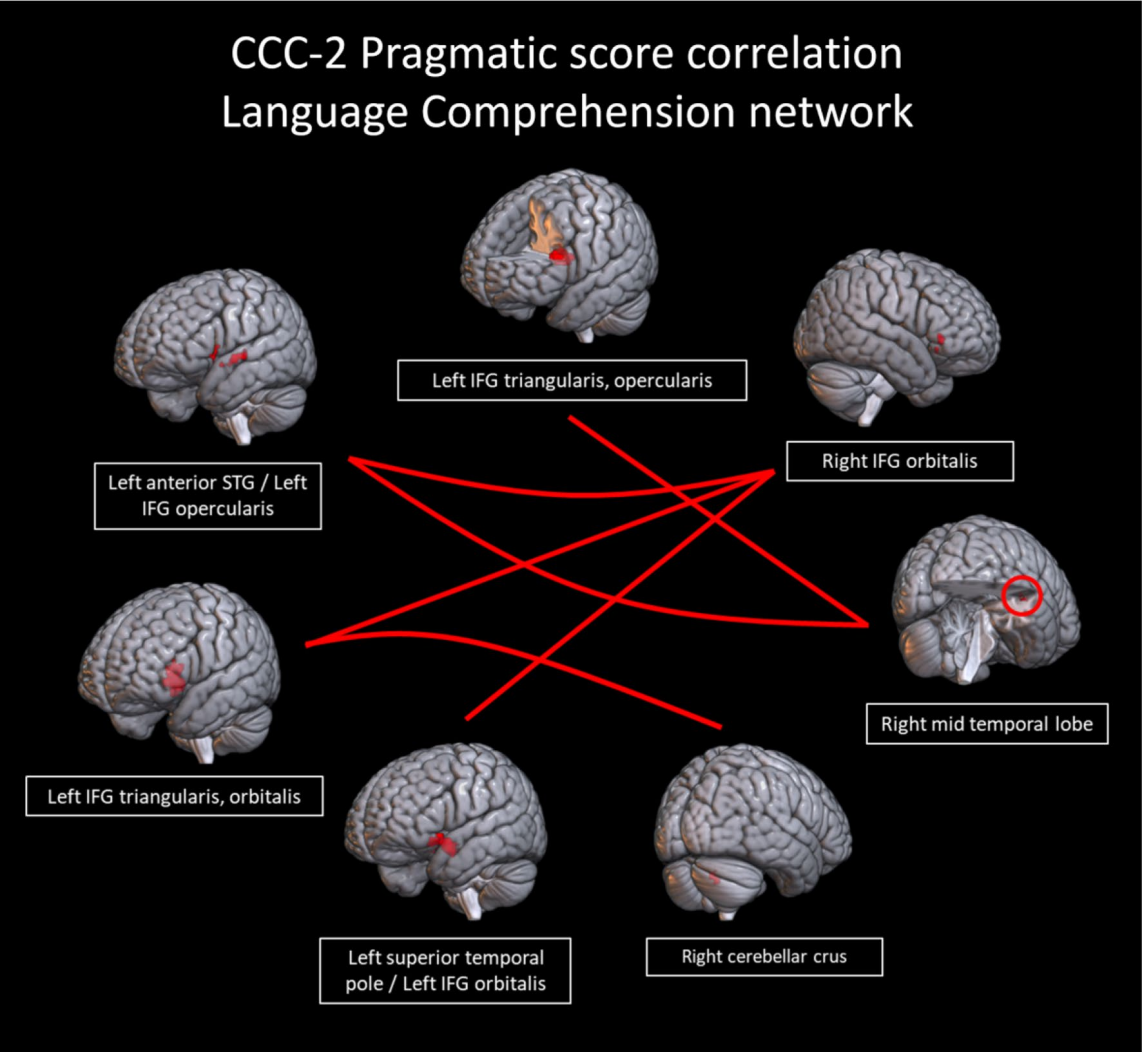
Connections between regions in the a priori language comprehension network from neurosynth that trended toward a positive correlation with CCC-2 pragmatic language scores (connection *p* < 0.005, cluster *p* < 0.1)

**Fig. 9:**
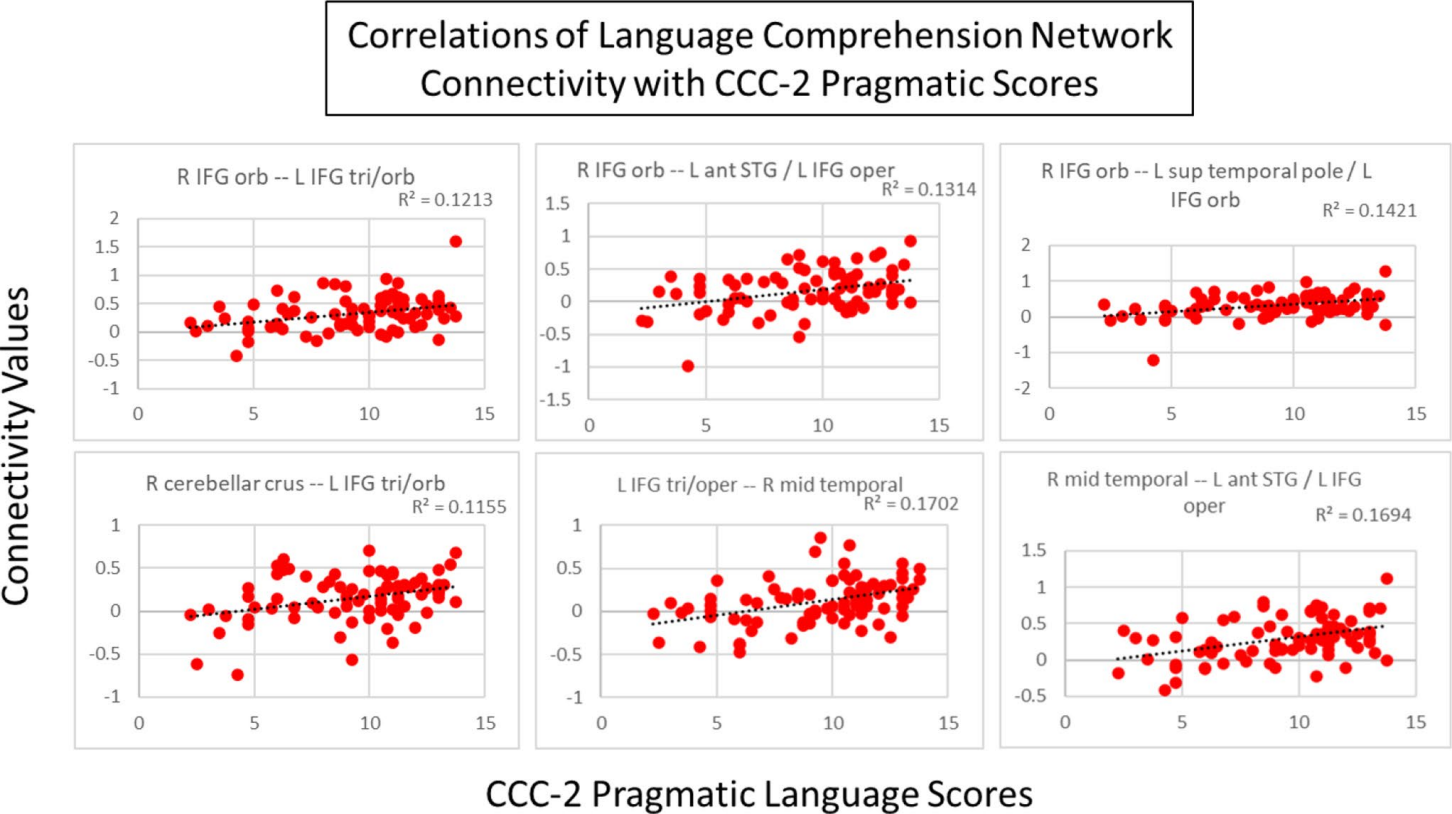
Scatterplots showing the correlation of the connectivity in the language comprehension network with CCC-2 pragmatic language scores (all r^2^ values displayed on graph, connection *p* < 0.005, cluster *p* < 0.1)

**Fig. 10:**
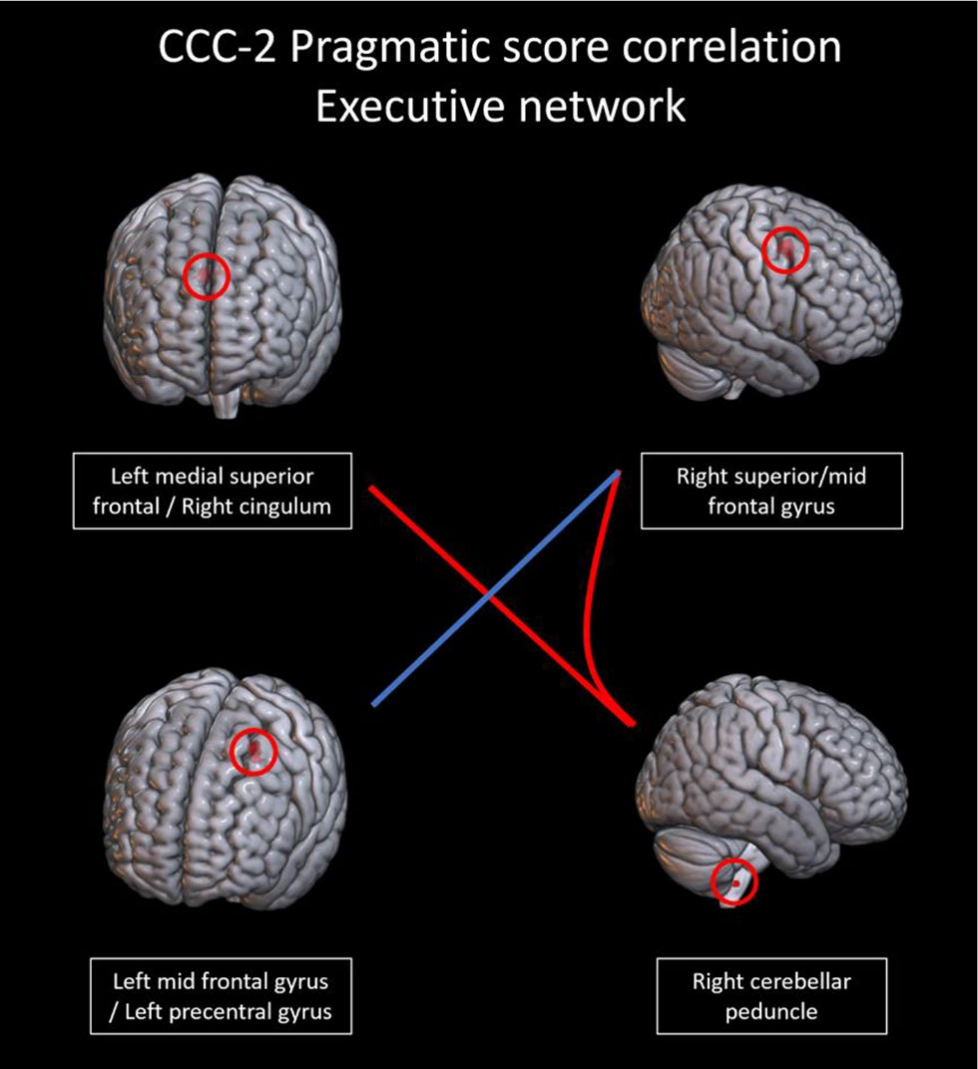
Connections between regions in the a priori executive network from neurosynth; red connections trended toward a positive correlation with CCC-2 pragmatic scores; the blue connection trended toward a negative correlation with the pragmatic scores (connection *p* < 0.005, cluster *p* < 0.1)

**Fig. 11:**
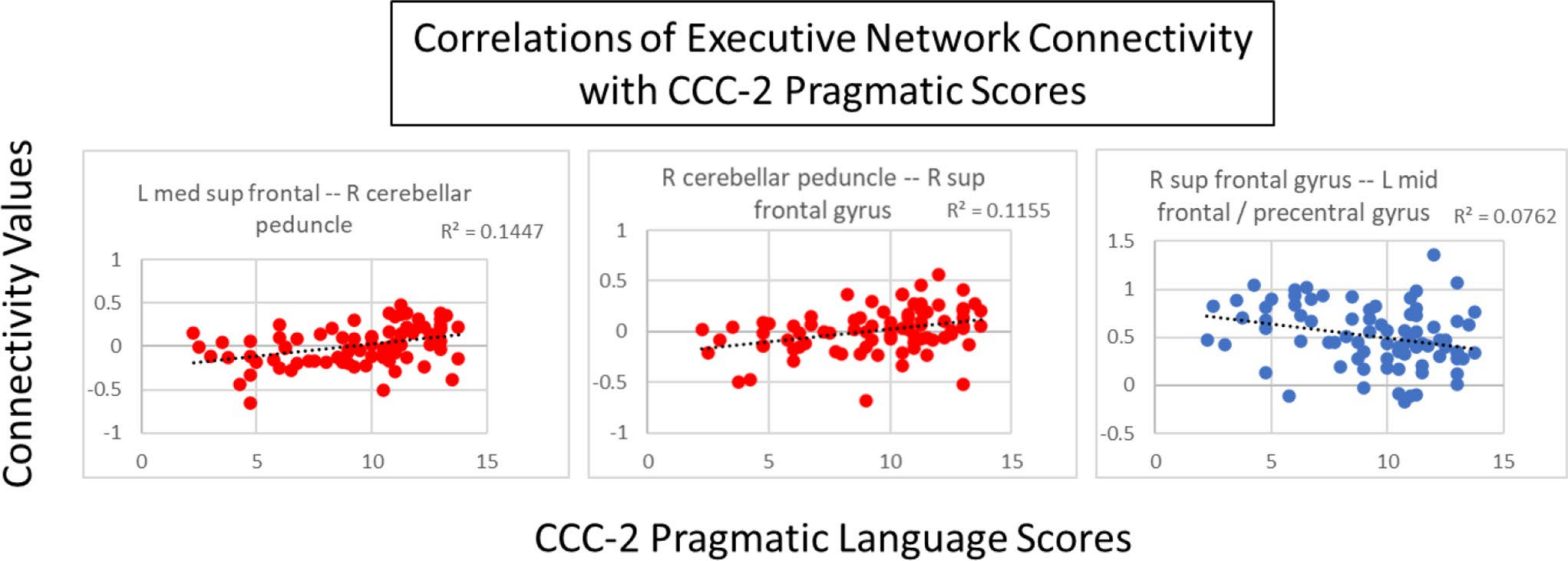
Scatterplots showing the correlation of the connectivity in the executive network with CCC-2 pragmatic language scores (all r^2^ values displayed on graph, connection *p* < 0.005, cluster *p* < 0.1). Blue shows negative correlations; red shows positive correlations

In the language comprehension and executive networks, several connections showed a trend toward correlations with pragmatic scores. In the language comprehension network, there was a trend toward a positive correlation (connection *p* < 0.005, cluster *p* < 0.1) with pragmatic scores in connections between portions of the left IFG and the following areas: right IFG, right temporal gyrus, and right cerebellar crus (Fig. 8). Figure 9 shows the correlations between connectivity and pragmatic scores. In the executive network, there was a trend (connection *p* < 0.005, cluster *p* < 0.1) toward a positive correlation with pragmatic scores in connections of the right cerebellar peduncle with the right STG and the left medial superior frontal gyrus, as well as a negative correlation with a connection between the left and right superior frontal gyri (Fig. 10). Figure 11 shows the correlations between connectivity and pragmatic scores.

Because of the high levels of correlation of CCC-2 subscale scores and NIH Toolbox cognition composite, we were unable to independently assess the separate contributions of these scores. Therefore, we chose not to include cognition composite scores as a covariate in our connectivity analyses, because of this high degree of collinearity with the language scores (Supplemental Table 1).

## Discussion

*Aim 1: Profiles of language skills based on CCC-2.* Our results showed that there were 33% of children in this study had lower skills in pragmatic or structural language or both. There was no pattern suggesting (or higher frequency of) deficits only in pragmatic or structural language (see Table 2).

The CCC-2 structural language subscale examines speech, syntax, semantics, and coherence skills. These include but are not limited to a child’s use of speech sounds, morphology, use of function words in context, vocabulary, and sequencing. The pragmatic language subscale examines initiation when speaking, nonverbal skills, social relations, and topical interests For example, these include whether it is difficult to stop the child from talking, whether the child looks at his or her interlocutor, shows anxiety when with other children, or shows unusual interests. Within our participants, these subscale scores were positively correlated. The CCC-2 is more widely known and used for its structural subscales, but many studies have also shown the effectiveness of the CCC-2 to gauge pragmatic language skills (Ferrara et al., 2020; Geurts & Embrechts, 2010; Stephan et al., 2021; Volden & Phillips, 2010). These studies have confirmed the specificity and sensitivity of the CCC-2 pragmatic subscales, but also suggest that it is ideal to use more than one measure or test of language skills, such as a combination of parent-report and child-facing, to provide more comprehensive information about the child’s language processing.

Although our study did not examine children with a diagnosed language disorder, the results on the scales of language skills have important implications for the assessment process. These findings suggest that difficulties in both structural and pragmatic language are likely to co-occur, and therefore should both be assessed in children with suspected language disorders. Currently, screening of speech and language delay or disorder is most often initiated by parent-report, regularly facilitated in school screenings, and focused on reading and verbal speech skills, sometimes with little to no emphasis on pragmatic skills (Bao et al., 2024). Often, pragmatic language is only considered when studying in the context of ASD (Law et al., 2013) but our findings indicate that even in a population without ASD, pragmatic language difficulties are observed with corresponding severity to structural language difficulties. The way in which structural language and pragmatic language influence each other can provide insight into how language develops both behaviorally and neurologically and warrants examination in future studies.

*Aim 2: Profiles of language skill associated with patterns of neural connectivity*. Our results showed that children who had both poorer pragmatic language skills and structural language skills had a trend toward increased connectivity between regions in the speech perception network, namely the bilateral STGs and the left Rolandic operculum. Although this hyperconnectivity across hemispheres, associated with poorer language skills, does not directly reflect a lower degree of left-lateralization, it is consistent with studies that show engagement of the right hemisphere in young children during language tasks, decreasing from childhood to adulthood (Everts et al., 2009; Holland et al., 2001, 2007; Olulade et al., 2020), but it is difficult to tell whether this particular inter-hemispheric hyperconnectivity represents a delayed neural maturation (Szaflarski et al., 2006) or the neural pattern of a language disorder (de Guibert et al., 2011), since the literature, especially the pediatric literature (see Weiss-Croft and Baldeweg (2015) for a review), is inconclusive (i.e. Krishnan et al. (2021) observed that children with DLD did not consistently show reduced lateralization).

*Connectivity and structural language scores*. Examining patterns of connectivity associated with language scores as a continuous variable allowed for more sensitive detection of the relationship between connectivity and language subscales. Due to the overlap of regions found in multiple ROI networks (see Fig. 1; Table 1), we did not complete a between-networks analysis. Not only did the original selected networks overlap but the results within each network shared some overlap. For example, connectivity involving the right STG was related to structural scores in the language comprehension network and the speech production network. Where these overlaps occurred, these regions exhibited unique connectivity with different regions in each network. For example, the right STG showed increased connectivity with the left fusiform gyrus in the language comprehension network, but decreased connectivity with the bilateral SMAs in the speech production network.

Within the language comprehension network, increased connectivity between the right STG and left inferior fusiform gyrus was associated with increased structural language ability. The left fusiform gyrus is considered to be the “visual word form area”. Children at the age included in our study have been shown to activate the visual word form area during reading (Fan et al., 2014; Li et al., 2017; Moulton et al., 2019; Saygin et al., 2016). And, in adults, the visual word form area supports the processing of written words (Dehaene et al., 2002; Gerrits et al., 2019; McCandliss et al., 2003). Children at the age included in our study have at least begun to develop reading skills, and this suggests that greater integration of visual word form processing with auditory processing and social cognition may be present in children with greater structural language skills. The role of the right STG in children is less clear, but in adults Luthra et al. (2023) discuss the likely functions of the right STG/STS as involved in the recognition of phonetic information for particular speakers, and in adolescents and adults, Rupp et al. (2022) discuss the role of the STG in voice encoding.

In the speech production network, connectivity also exhibited a trend in correlation with structural language ability. In the group comparison described above, children with combined language difficulties showed hyperconnectivity in this network, particularly in the left and cross-hemispheric connections between the temporal and inferior Rolandic areas. However, when examining structural language scores as a continuous variable, the pattern was more complex. Consistent with the results of the group comparison, structural language scores correlated negatively with connections between left and right Heschl’s gyrus, right Heschl’s gyrus and bilateral SMA, and right Heschl’s gyrus to right IFG. This suggests that connections between primary auditory cortex and regions involved in motor planning (i.e. the dorsal pathway) exhibit hyperconnectivity associated with lower structural language scores.

However, structural scores also trended toward a positive correlation with a number of connections in the speech production network both within and between hemispheres. Connections including subcortical regions such as the right putamen and left thalamus, as well as between the cerebellum to right STG were increased in children with higher structural language scores. This suggests that these skills are also supported by connections among regions outside of the ventral and dorsal streams of language processing. Subcortical regions, shown previously to have reduced fMRI activation in children with SLI (Badcock et al., 2012; de Guibert et al., 2011) support multisensory integration, learning, reward, and motor control including articulation and language processing (Vinas-Guasch & Wu, 2017), and the cerebellum has a recognized role in speech and language (Vias & Dick, 2017), and reading (Hutton et al., 2017). Though Ullman et al. (2024) have considered the putamen to be involved in procedural learning aspects of language due to its involvement in automatization of procedures (Doyon et al., 2009; Janacsek et al., 2020; Tagarelli et al., 2019; Ullman, 2004; Ullman et al., 2020) and (especially of anterior portions) its link to the cognitive and language impairments of DLD (Arsalidou et al., 2013; Crosson et al., 2003; Janacsek et al., 2022; Ullman & Pierpont, 2005; Vinas-Guasch & Wu, 2017). Previous findings suggest that the putamen assisted with the automatization of sequential motor movements needed for speech (Krishnan et al., 2016) and had lower connectivity with other subcortical structures such as the hippocampus and temporal pole in those at high risk for dyslexia (Hosseini et al., 2013), but later was found that there are no consistent activation differences in the putamen when comparing children with DLD and TD children (Krishnan et al., 2021). Our results adhere with the possibility that the putamen and other subcortical regions are responsible for processing the structural components of language.

Increased connectivity between a region of the left occipital lobe with bilateral SMA and with right IFG was associated with higher structural language scores. There has been limited prior work in children related to the role of occipital regions in language processing, but connections between occipital and other cortical regions may reflect the ability to visually track the face of an interlocutor, visual imagery associated with speech and language (Ashburn et al., 2023; Turker et al., 2023), or reading skills (see Wang et al. (2023), though this study examined this only in the left hemisphere). The occipital lobe has several functions and communicates readily with the visual word form area in children (Ben-Shachar et al., 2011) and in adults (Devlin et al., 2006; Reinke et al., 2008; Xue et al., 2006; Xue & Poldrack, 2007). Notably, the ventral occipital cortex is engaged during reading in children (Ben-Shachar et al., 2011; Lerma-Usabiaga et al., 2018; Olulade et al., 2013; Richlan et al., 2010; van der Mark et al., 2011) and adults (Cohen et al., 2004; Leff et al., 2006; Xue et al., 2006) and is engaged in relation to phonological awareness in children (Pleisch et al., 2019; Wang et al., 2018, 2021). 

*Connectivity and pragmatic language scores*. Within the language comprehension network, a trend in increased connectivity among the bilateral IFGs, right temporal gyrus and right cerebellum were associated specifically with pragmatic language ability, suggesting that these connections contribute to higher-level language skills. Previous studies have not directly examined the relationship between connectivity and pragmatic language skills in children, but our results are consistent with finding in the adult literature, or pediatric studies focused on other language domains. The association of the cerebellum with pragmatic language ability adds to previous pediatric studies that have found increased activation of the cerebellum related to greater interest and engagement in reading (Hutton et al., 2017) and activation of the cerebellum during phonological tasks (Turker et al., 2023). Damage to the cerebellum and basal ganglia can result in poorer language skills in adults (Silveri, 2021) and functional abnormalities in the basal ganglia and parietal lobe are associated with DLD (Ullman et al., 2024). Our results indicate that regions of the brain associated with structural language processing may play a greater role in the higher-order processing needed for pragmatic language skills than previously expected. These functional connections, particularly between bilateral frontal and temporal regions may play a role in interpreting auditory information not merely for speech content, but for the broader social and emotional contexts the language occurred in. These connections, suggested to support pragmatic language skills, include aspects of both the dorsal and ventral streams of the dual-stream model (Friederici, 2012), functionally connecting temporal regions that project to the pars opercularis of the IFG. The IFG, bilaterally, has been suggested to support the procedural memory component of the declarative/procedural model (Ullman, 2004), specifically, rule-based aspects of language skill that may include pragmatic processes such as inference and discourse comprehension and production.

Pragmatic language scores also correlated with connectivity in the executive network. Some connections correlated positively with pragmatic scores – specifically, connections of the right cerebellar peduncle with medial and right superior frontal regions and the right cingulum. One connection correlated negatively with pragmatic scores – a connection between the left and right superior frontal gyri. While the neural basis of pragmatic language skills has not been established in children, in adults, these areas are involved in higher order processing and executive function (Braunlich et al., 2015; Clemens et al., 2014; Noonan et al., 2013). Dibbets et al. (2006) also found that frontal regions associated with executive function were uniquely engaged in children with SLI. Executive function impacts pragmatic language particularly as it relates to social cognition (Andres-Roqueta et al., 2021) and notably in children with ASD (Razavi et al., 2019). Level of pragmatic skills may be related to degree of connectivity between cerebellar and frontal regions, perhaps indicating a role of the coordination of speech production, sequencing, and higher order processing in children (Rupp et al., 2022; Vias & Dick, 2017; Wang et al., 2023).

Our results also align with the literature on the neural correlates of pragmatic language processing in adults, though these studies have focused on task-related areas of activation, rather than the connections between them. A number of tasks have been used with the goal of engaging pragmatic language skills. For example, de Almeida et al. (2016) found activation in the left inferior frontal gyrus, ventromedial pre-frontal cortex, the bilateral superior temporal gyri, and the anterior cingulate cortex in response to participants listening to ambiguous sentences. Basnakova et al. (2014), using an auditory inference comprehension task found activation in the bilateral superior medial frontal gyri, the pars orbitalis and pars triangularis of the inferior frontal gyri, and the right supplemental motor area when participants were listening to sentences requiring socio-emotional inference compared to those that did not. Similarly, our results found both hyper- and hypo-connectivity related to the bilateral SMAs. Shibata et al. (2010) observed the neural correlates of irony comprehension and found activation in ROIs that correspond with our results, including the inferior frontal gyri, the right superior frontal gyrus, and the middle frontal gyrus. They conclude that these areas of activation may indicate the influence of higher-order cognitive processing on pragmatic processing, akin to our results associated with pragmatic language skills in the frontal regions of the executive network. In relation to those with autism spectrum disorder, Tesink et al. (2009) compared participants listening to sentences that were either congruent or incongruent in content and speaker identity. They found increased activity in the ASD group in the right inferior frontal gyrus for the incongruous condition despite no behavioral differences when compared to typically developing peers. The bilateral inferior frontal gyri may also play a role in comprehending prosody, needed for pragmatic language processing (Beaucousin et al., 2007; Witteman et al., 2012) much like our connectivity results in the same regions of the language comprehension network.

Some of the limitations of this study include the recruitment criteria and use of Neurosynth ROI maps. Since the participants were recruited due to primary concerns related to, this caused the sample to be more likely to have language difficulties than a truly random sample would. While the use of Neurosynth has several advantages detailed previously, the maps produced are averaged maps of both pediatric and adult data; ROI maps created solely on pediatric data would be ideal. The resting-state data were also limited, not only due to participant success in the scanner, but also do to the relatively short duration of the scan. Lastly, if we had task-based fMRI to compare activation in our same ROI networks to resting state connectivity, this would further validate our resting state results. Notably, if the in-scanner tasks included distinctly pragmatic and structural tasks.

## Conclusion

The results of this study indicated that structural and pragmatic language skills were associated with altered patterns of functional connectivity including regions such as the bilateral inferior frontal gyri and superior temporal gyri, Heschl’s gyrus, areas including bilateral supplementary motor areas, and parts of the cerebellum. As we hypothesized, in part, pragmatic scores correlated with connectivity within the executive and language comprehension network, but not in the speech networks. Likewise, as partially anticipated, structural scores correlated with connectivity in the speech production network, but also with connectivity in the language comprehension network. By relating specific aspects of language skill to patterns of functional connectivity, this work represents a next step in identifying neural substrates of specific profiles of language difficulty in school-age children. There are many future directions that can be taken given these results. The CCC-2, as a brief parent-report measure, could be complemented through child-facing assessments of specific aspects of structural and pragmatic language processing. Given the paucity of literature on the neural basis of pragmatic language in children, it would be of interest to further investigate aspects of pragmatic language including inference, topic maintenance, and emotion processing. This would require a broader range of child-facing assessments. Also, using task-based fMRI to assess the neural basis of structural and pragmatic language skills, and how they interrelate in school-age children, would allow investigation of whether differences observed in resting state networks parallel differences in those networks during task activation. In general, expanding our behavioral and neuroimaging approaches to include pragmatic language skills allows for a more comprehensive profile that better reflects children’s real-world language use.

## Electronic supplementary material

Below is the link to the electronic supplementary material.

Supplementary Material 1

## Data Availability

Data will be made available upon request.

## Footnotes

1 We note that most of these earlier studies use the term “Specific Language Impairment” to describe individuals with diagnosed language difficulties; we recognize that more recent work (Bishop et al., 2016; Bishop et al., 2017) has clarified this terminology, and “Developmental Language Disorder” may be more appropriate depending on the participants’ profiles.
