# Supplementary material for: Structural and pragmatic language skills in school-age children relate to resting state functional connectivity
Source: Brain Imaging Behav. 2025 Jul 8;19(6):1146–67. doi: 10.1007/s11682-025-01040-7 (PMC12831709; doi:10.1007/s11682-025-01040-7)
Supplement: Supplementary file 1 — Supplementary Material 1 [file 11682_2025_1040_MOESM1_ESM.docx]

**Supplementary Materials**

|  | NIH Toolbox Cognition Composite scores |
| --- | --- |
| CCC-2 Structural Language scores | Pearson r = 0.638 p<0.001 |
| CCC-2 Pragmatic Language scores | Pearson r = 0.398 p<0.001 |

**Supplemental Table 1.** Correlation matrix of CCC-2 subscale scores and NIH Toolbox cognition composite.
